# The Effectiveness of Ultrasound-Guided Infiltrations Combined with Early Rehabilitation in the Management of Low Back Pain: A Retrospective Observational Study

**DOI:** 10.3390/diagnostics14182087

**Published:** 2024-09-20

**Authors:** Danilo Donati, Fabio Vita, Vincenza Amoruso, Flavio Origlio, Roberto Tedeschi, Francesco Castagnini, Salvatore Massimo Stella, Marco Miceli, Cesare Faldini, Stefano Galletti

**Affiliations:** 1 Physical Therapy and Rehabilitation Unit, Policlinico di Modena, 41125 Modena, Italy; danilo.donati@unimore.it; 2 Clinical and Experimental Medicine PhD Program, University of Modena and Reggio Emilia, 41125 Modena, Italy; 3 IRCCS Istituto Ortopedico Rizzoli, 1st Orthopaedics and Traumatology Clinic, 40136 Bologna, Italy; 4 Rehabilitation Unit Santa Corona Hospital, 17027 Pietra Ligure, Italy; 5 Physical Therapy and Rehabilitation Unit, IRCCS Rizzoli Orthopedic Institute, University of Bologna, 40136 Bologna, Italy; 6 Department of Biomedical and Neuromotor Sciences, Alma Mater Studiorum, University of Bologna, 40136 Bologna, Italy; 7 SC Ortopedia e Traumatologia e Chirurgia Protesica e dei Reimpianti di Anca e Ginocchio, IRCCS Istituto Ortopedico Rizzoli, 40136 Bologna, Italy; 8 SIUMB Advanced School for Musculoskeletal Ultrasound, Department of Clinical and Experimental Medicine, University Post-Graduate Course, Santa Chiara University Hospital, 56121 Pisa, Italy; 9 IRCCS Istituto Ortopedico Rizzoli, Diagnostic and Interventional Radiology, 40136 Bologna, Italy; 10 Musculoskeletal Ultrasound School, Italian Society for Ultrasound in Medicine and Biology, 40136 Bologna, Italy

**Keywords:** ultrasound therapy, rehabilitation, chronic pain, musculoskeletal disorders, pain management

## Abstract

**Background and Aims:** Low back pain is a prevalent condition affecting 60–85% of individuals during their lifetime. Despite various proposed mechanisms, the etiology of low back pain remains unclear. This study aims to evaluate the effectiveness of combining ultrasound-guided infiltrations with early rehabilitation in reducing pain and improving functional limitations in patients with chronic nonspecific low back pain. **Methods:** A retrospective observational study was conducted, reviewing data from January to April 2024 involving 40 patients with chronic nonspecific low back pain. Each patient received two cycles of ultrasound-guided lidocaine and corticosteroid infiltrations at the level of the posterior lower iliac spine, followed by 10 rehabilitation sessions. Patients were assessed at baseline (T0), after the first treatment cycle (T1), and after the second cycle (T2) using the Oswestry Disability Index, Quebec Back Pain Disability Scale, Roland Disability Questionnaire, and Numeric Rating Scale. **Results:** Significant improvements were observed across all assessment scales. The ODI scores decreased from 33.5 at baseline to 3.5 after treatment (*p* < 0.001). Similar reductions were noted in the QBPDS (from 61.5 to 10.3), RDQ (from 18 to 3.4), and NRS (from 7.4 to 1.3). The combination of ultrasound-guided infiltrations and early rehabilitation resulted in a significant reduction in pain and disability, with the most notable improvements occurring after the second treatment cycle. **Conclusions:** The integration of ultrasound-guided infiltrations with early rehabilitation is highly effective in managing chronic nonspecific low back pain, significantly reducing both pain and functional limitations.

## 1. Introduction

Low back pain is a significant musculoskeletal issue that substantially impairs functional abilities and diminishes the quality of life, particularly among the elderly population. Recently, the United Nations highlighted the critical impact of low back pain, acknowledging it as one of the primary contributors to disability in individuals aged 60 and above. This condition not only imposes severe physical limitations but also exerts considerable economic and social burdens. Affecting over 80% of adults at some point in their lives, low back pain stands as the leading cause of disability worldwide, underscoring the urgency for effective management strategies [1]. Low back pain is described as pain localized between the 12th rib and the gluteal region, with or without irradiation to the lower extremities. The critical burden of low back pain was highlighted by a recent study showing its impact on older adults [2]. Low back pain covers a spectrum of different types of pain (e.g., nociceptive, neuropathic and nociplastic, or nonspecific) that often overlap [1,3].

The role of connective tissues, particularly that of the thoracolumbar fascia, has been studied extensively. Deep fascia is a three-dimensional network of connective tissue that envelops bones, muscles, nerves, and blood vessels throughout the body. Recently, its importance in the persistence of debilitating conditions, such as back pain, has been emphasized [4,5,6]. Fascia is a type of connective tissue composed of irregularly arranged collagen fibers. This arrangement enables it to effectively withstand tension forces, but it is less suited to withstand compression forces. The thoracolumbar fascia is created by the fusion of the aponeurotic and fascial planes that form the retinaculum around the muscles of the lumbar and sacral region [7]. Its role is to distribute and balance forces between the spine, pelvis, and lower limbs during standing and walking. The thoracolumbar fascia plays a vital role in supporting the spine and maintaining proper posture by coordinating with the spinal muscles [8]. Recent studies have increasingly recognized the role of the thoracolumbar fascia in chronic low back pain, highlighting its contribution to the persistence of pain symptoms [7,9].

The management of low back pain involves a combination of nonpharmacological and pharmacological treatments. The first line of treatment typically focuses on nonpharmacological options, including therapies supported by various studies, such as counseling, structured exercise programs, spinal manipulation, massage therapy, heat application, dry needling, acupuncture, transcutaneous electrical nerve stimulation (TENS), and physical therapy [10,11]. These physical therapy and exercise programs have shown benefits in both the medium and long term. When nonpharmacological measures are insufficient, pharmacological treatments are considered as secondary options. Nonsteroidal anti-inflammatory drugs (NSAIDs) are usually the first choice, with duloxetine also offering potential benefits. While the evidence regarding the routine use of corticosteroids remains inconclusive, corticosteroid injections have been effective in some cases of low back pain. Surgical intervention is generally not required for the majority of chronic low back pain patients; however, it may be necessary for those with significant functional impairments or persistent pain caused by conditions such as progressive spinal stenosis, worsening spondylolisthesis, or herniated discs [10]. Preventative measures are crucial, especially when managing acute back pain, as early intervention can reduce the risk of developing chronic conditions [12]. Utilizing screening tools to identify those at risk of progression from acute to chronic pain can help in applying targeted treatment strategies, thereby preventing the escalation of symptoms [10,13,14]. Thus, considering the known treatment strategies, this study aims to investigate the effectiveness of the combination of physical and infiltrative therapy for the treatment of low back pain. This retrospective observational study reviews patient data to evaluate the effectiveness of combining ultrasound-guided infiltrations with early rehabilitation.

## 2. Material and Methods

The study received approval from our institutional review board.

All participants provided informed consent in accordance with our institution’s data collection and disclosure policy. Further ethical review was deemed unnecessary as no personally identifiable information was collected or stored.

This was a retrospective observational study, analyzing data from patients referred to the Physical Medicine and Rehabilitation Unit who underwent ultrasound assessment for chronic low back pain between January and April 2024. Thus, patients between 18 and 65 years, who had pain symptoms lasting more than 3 months, with at least 1 pain episode per year in the past 2 years, were included in the study. Conversely, patients with a history of spinal surgery, spinal deformity, or vertebral fractures, and who refused to sign informed consent, were excluded (Table 1).

Patients included in the study were evaluated at the beginning of the treatment protocol (baseline, T0), after 5 weeks (T1), and at the end of the protocol, after 10 weeks (T2). Age, sex, body mass index (BMI), and duration of algic symptoms were recorded at T0 for each patient. Patients also underwent clinical assessment for each time point using the Italian version of the Oswestry Disability Index (ODI-I) [15], the Roland Morris Disability Questionnaire (RMDQ) [16], the Quebec Back Pain Disability Scale (QBPDS) [17], and the Numeric Rating Scale (NRS) [18].

The ODI-I is a questionnaire consisting of 10 sections, each of which contains within it 6 possible answers with scores ranging from 0 to 5, where 0 corresponds to no difficulty or pain and 5 corresponds to an inability to perform the activity or disabling pain. Each section investigates the impact of low back pain on different aspects of daily life, such as pain intensity, personal hygiene, the ability to lift weights, walk, sit and stand, sleep, social life and sex life, and finally, the ability to travel. By filling in all sections, a maximum score of 50 points can be obtained, and by using the formula $\left(\frac{score\;for\;each\;section}{total\;score}\right)\times 100$, the percentage score can be obtained. Considering the latter, 5 classes of disability can be identified: 0–20% for minimal disability, 21–40% for moderate disability, 41–60% for severe disability, 61–80% for severe disability, and 81–100% for complete disability [19]. The RMDQ is a self-administered rating scale consisting of 24 questions to investigate the patient’s ability or limitation in performing 24 activities. Each activity can be given a score of 0 if there are no limitations or 1 if there are difficulties due to back pain. The total score is between 0 and 24 [19]. The QBPDS is a self-assessment tool for low back pain through 20 items. For each item, a score ranging from 0 (no difficulty) to 5 (maximum difficulty) can be assigned. The total score is calculated by summing the scores of each item, resulting in a score ranging from 0 (no disability) to 100 (maximum disability). The NRS is a one-dimensional, 11-point rating scale that assesses pain intensity, where 0 corresponds to “no pain” and 10 corresponds to “worst pain imaginable”. The NRS has been widely used in clinical settings for pain assessment [18].

### 2.1. Infiltration Dosage

We performed ultrasound-guided infiltrations using a mixture of 2 mL of lidocaine 2% and 1 mL of corticosteroid (methylprednisolone 40 mg/mL). The infiltration was administered at the level of the postero-superior iliac spine for each patient. This dosage was maintained consistently across all patients to ensure reproducibility of results. Care was taken to monitor the administration and ensure that the dosage was accurately delivered under ultrasound guidance.

### 2.2. Treatment Protocol for Low Back Pain

**Figure 1:**
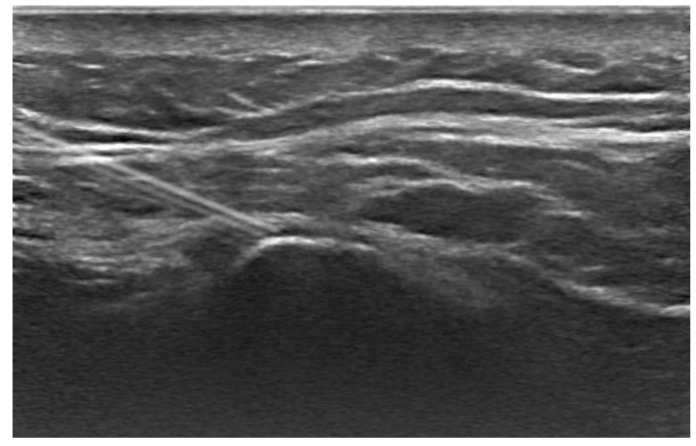
Ultrasound-guided injection. The image shows an ultrasound-guided injection procedure with the needle precisely positioned near the spine. This technique is used to deliver medication directly to the affected area, reducing pain and improving functionality in patients with chronic low back pain.

The treatment protocol adopted at the Physical and Medicine Rehabilitation Unit stipulated that each patient, after undergoing an initial assessment at baseline (T0), underwent an echo-guided infiltration (Figure 1) with lidocaine and corticosteroids at the level of the postero-superior iliac spine, and then a rehabilitative treatment of 10 sessions of 30 min each, biweekly. At the end of the first 5 weeks of treatment (T1), the patients underwent an evaluation with the clinical scales described above and a new ultrasound-guided infiltration of lidocaine and corticosteroids, followed again by a cycle of 10 rehabilitation sessions according to the scheme described above. Subsequently, patients underwent another cycle of lidocaine infiltration and corticosteroids, followed by a 10-session physical therapy program (twice a week). At the end of the second course of treatment, patients were evaluated using the rating scales described above.

Regarding rehabilitation treatment, the protocol for low back pain that all the patients underwent consisted of 6 stages. Abdominal strengthening: The exercise involved flexing the hip by bringing the knee toward the chest as far as possible, without lifting the pelvis off the table, and remaining in this position for 10 s before returning to the starting position. The exercise was repeated 10 times, alternating lower limbs.

Pelvis retroversion: The exercise involved pressing the back against the couch while contracting the abdominal and gluteal muscles, holding the position for 10 s, and then returning to the starting position. The exercise was repeated 10 times.

Supine postural correction: The patient was asked to crush the lumbar lordosis using contractions of the abdominal and gluteal muscles, hold the position for 10 s, and then return to the starting position. The exercise was repeated 10 times.

Stretching of the gastrocnemius muscles: The patient, positioned standing facing the wall with their hands resting on the wall, with one leg extended, and the other flexed, was asked to bend toward the wall while keeping their back straight, hold the position for 10 s, and then return to the starting position. The exercise is repeated 10 times.

Strengthening the buttocks: The exercise involved resting the torso on the couch (or on a table) with the hips flexed at 90° and the feet on the floor, and slowly and alternately raising the lower limbs, without arching the lumbar region and keeping the torso and head in contact with the couch and holding this position for 10 s and then returning, slowly, to the starting position. The exercise was repeated 10 times per leg.

**Figure 2:**
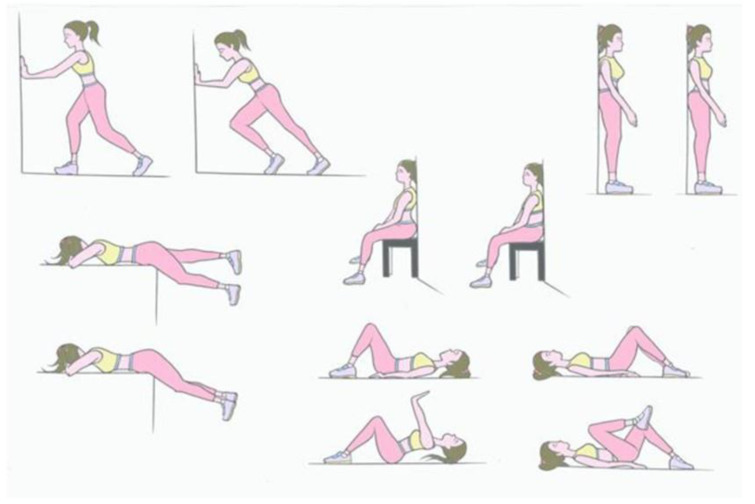
Postural rehabilitation exercises. A series of postural rehabilitation exercises aimed at strengthening the abdomen, aligning the spine, and improving posture. These exercises are an integral part of the rehabilitation protocol for patients with chronic low back pain, helping to reduce pain and enhance mobility.

Standing postural correction: The patient was asked to lean with their shoulders against a wall with their feet about 30 cm from the wall and to level the lumbar lordosis (i.e., to eliminate the space between the wall and the back) by contracting the abdominal and gluteal muscles, and to hold this position for 10 s and then return to the starting position. The exercise was repeated 10 times (Figure 2.)

The study design follows the STROBE guidelines for observational studies. All patients were evaluated based on predefined inclusion and exclusion criteria. The treatment protocol was standardized, and outcomes were assessed using validated tools, such as the Oswestry Disability Index and the Numeric Rating Scale.

### 2.3. Statistical Analysis

Considering the study design, no formal sample size estimation was performed. Results were reported as difference-in-differences (MD) with 95% confidence intervals (CI) and *p*-values.

Time trends of the ODI, NRS, QBPDS, and RDQ were estimated using multivariable repeated measures mixed models. In these models, the effect of time was adjusted for age, sex, BMI, and type of work (office or manual). A random intercept term was also included in the models to handle the correlation between models and the correlation between repeated measures.

### 2.4. Sample Size Considerations

As this was a retrospective observational study, no formal sample size calculation was performed a priori. However, based on the observed effect size in this study (a mean difference of 6.1 on the Numeric Rating Scale [NRS] with a standard deviation of 5.2), a post hoc power analysis was conducted. Using a two-tailed test with an alpha level of 0.05 and aiming for 80% statistical power, the estimated minimum sample size required to detect a clinically meaningful difference would have been 6 patients. Given the study’s sample size of 40 patients, the sample was deemed adequate to capture the observed effect sizes with sufficient power.

## 3. Results

In the time interval from 1 January 2024 to 30 April 2024, a total of 40 patients were ultrasound-assessed for chronic nonspecific low back pain. They were treated with infiltration with lidocaine and corticosteroid (DOSE) and a rehabilitation cycle of 10 sessions (twice a week).

**Figure 3:**
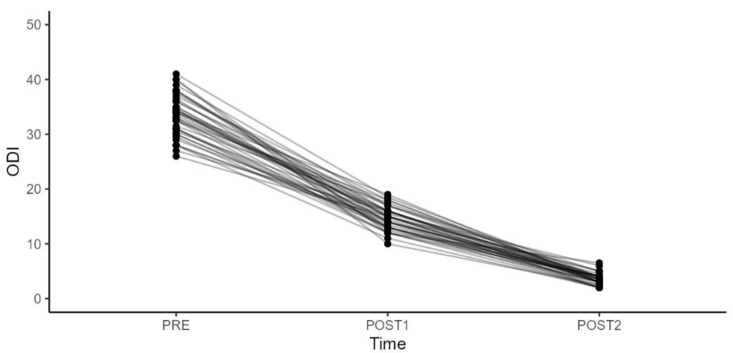
Oswestry Disability Index (ODI) scores during different treatment periods and comparison between variables.

**Figure 4:**
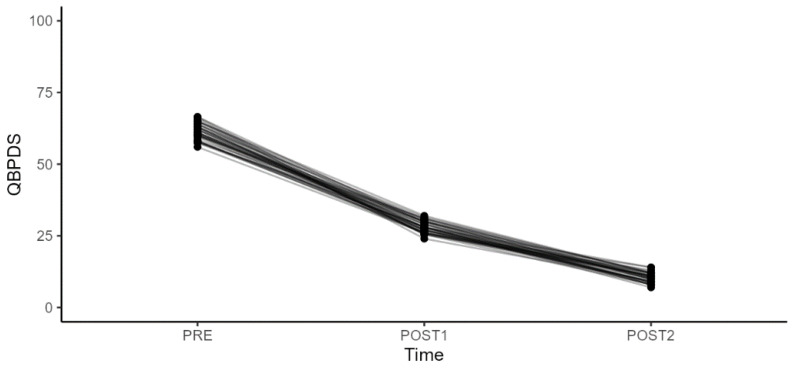
Quebec Back Pain Disability Scale (QBPDS) scores during different treatment periods and comparison between variables.

**Figure 5:**
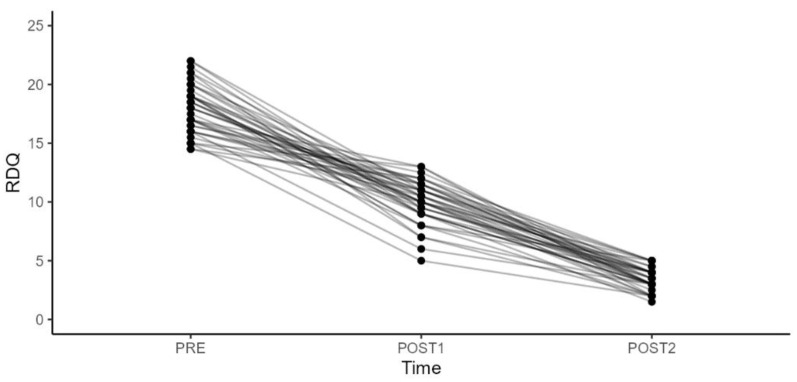
Roland Disability Questionnaire (RDQ) results during various observation periods and for the analyzed variables.

**Figure 6:**
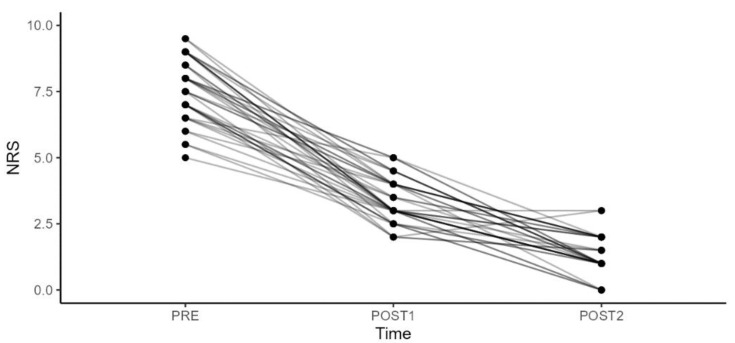
Numeric Rating Scale (NRS) trends for pain levels following treatments.

The patients, including 16 men and 24 women, had a mean age of 41.5 years (min 18–max 65). Functional outcomes were also rigorously assessed using the Oswestry Disability Index (ODI), Quebec Back Pain Disability Scale (Qbpds) and Roland Disability Questionnaire (RDQ) (Table 2, Table 3, Table 4 and Table 5) (Figure 3, Figure 4, Figure 5 and Figure 6).

We observed a statistically significant improvement (*p*-value < 0.001) in the Oswestry Disability Index (ODI) scores when comparing the period between the second rehabilitation cycle and infiltration (T2) and the first rehabilitation cycle and infiltration (T1), and at baseline T0, as well as between T2 and T0 (Table 2). The most notable improvement was seen between T2 and T0, with a mean difference value of −30.

However, we found no statistically significant differences in the ODI changes related to sex, BMI, age, and work, either between T1 and T0, between T2 and T0, or between T1 and T2.

### Statistical Power

A post hoc power analysis was conducted based on the sample size of 40 patients, an observed effect size of 6.1 on the Numeric Rating Scale (NRS), and a standard deviation of 5.2. The analysis revealed a statistical power of 99.9%, indicating that the sample size was sufficient to detect the observed effect with a high level of confidence.

Regarding the QBPDS, we observed a significant improvement (*p*-value < 0.001) in the scale values when comparing the period between the second rehabilitation and infiltration cycle (T2) and the first rehabilitation and infiltration cycle (T1), as well as at baseline T0, and between T2 and T0. The most noticeable improvement occurred between T2 and T0, with a mean difference value of −51.2.

As for changes in the ODI concerning sex, BMI, age, and work, there were no statistically significant differences either between T1 and T0 or between T2 and T0.

In terms of the RDQ, there was a statistically significant improvement (*p*-value < 0.001) in the scale values when comparing the period between the second rehabilitation cycle and infiltration (T2) and the first rehabilitation cycle and infiltration (T1), as well as at baseline (T0) and between T2 and T0. The most significant improvement was observed between T2 and T0, with a mean difference value of −14.6. On the other hand, there were no statistically significant differences in the ODI changes related to sex, BMI, age, and work, either between T1 and T0 or between T2 and T0.

We observed a significant improvement in the NRS scale values (Table 5). The improvement was statistically significant when comparing the period between the second rehabilitation cycle and infiltration (T2) and the period between the first rehabilitation cycle and infiltration (T1), as well as when comparing with the baseline at T0, and between T2 and T0. The most notable improvement was seen between T2 and T0, with a mean difference value of −6.3. Regarding changes in the ODI in relation to gender, BMI, age, and work, we found no statistically significant differences either between T1 and T0 or between T2 and T0.

Initially, the collective baseline ODI score for the group pre-treatment was 33.5 ± 1.3, indicating a moderate to severe disability level prior to any treatment. Following the treatment, a substantial reduction in disability was observed, with the mean ODI score decreasing dramatically to 3.5.

In addition, the scales used to assess the disability and pain of patients showed similar results: the RDQ decreased from 18 ± 7.2 before the treatment to 3.4 ± 5.6; the QBPDS from 61.5 ± 7.4 to 10.3 ± 3.5; and the VAS from 7.4 ± 5.2 to 1.3 ± 2.5.

## 4. Discussion

The present study aimed to demonstrate how the combined treatment of ultrasound-guided infiltration together with early rehabilitation treatment can contribute to the significant reduction to resolution of low back pain and improvement in ADL limitations. Ultrasound-guided injections have been shown to provide more precise delivery of medication, leading to better outcomes in the management of chronic low back pain [20,21,22].

Low back pain (LBP) stands as the most common musculoskeletal issue affecting people today. Over the past 30 years, clinical approaches to managing LBP have evolved, with a growing emphasis on enhancing function through improved activity and participation rather than solely focusing on pain relief. This shift towards rehabilitation is driven by the realization that interventions aimed only at alleviating pain have not been effective in significantly reducing the overall impact of low back pain on the population [23]. Early rehabilitation, particularly when combined with physical therapy, has been shown to prevent the progression from acute to chronic low back pain [24]. The clinical practice guidelines recommend multimodal approaches to manage low back pain; in our study, we decided to combine physical therapy and infiltration intervention [25]. Our study of 40 patients demonstrated that the combination of physical therapy and infiltrative treatment led to improved back disability and reduced pain intensity [26,27]. The results indicated a significant decrease after the second treatment cycle, suggesting that these positive outcomes are a result of the intensive treatment provided to the patients. Concerning the results of the Oswestry Disability Index (ODI-I), which is a scale that evaluates the intensity of pain and the disabling effect of pain on typical daily activities, from our results, all patients had a statistically significant decrease, with a *p*-value < 0.001, of the ODI-I values both after the first treatment cycle with a mean difference of −18.8, and after the second treatment cycle with a mean difference of −30.0. From the results, we see that the combined treatment in the time between T0 and T2 results in a decrease in pain and a greater benefit on functional autonomy than after only one cycle. At the same time, there seems to be no difference in the ODI trend in correlation with sexual gender, BMI, age, or type of work performed. We obtained similar results regarding the performance of the QBPDS. This scale assesses the impact of low back pain on back disability across 20 items. After the first treatment cycle, we observed a decrease in back disability caused by pain with a mean difference of −28.4. These decreases increased after the second treatment cycle to −51.20 for the RDQ, where the mean difference between pre- and post-treatment increased from −8.0 to −14.6. Additionally, for the NRS, the mean score increased from 4.24 to 6.24 W. After the first and second treatment cycles, we observed the following mean differences in the three rating scales: −11.20 for the ODI, −22.8 for the QBPDS, and −6.60 for the RDQ. These results indicate a consistent improvement in back pain and disability after the second treatment cycle. The greater decrease in the QBPDS rating scale compared to the others may be due to its larger number of items than the ODI. Additionally, compared to the RDQ, the QBPDS shows greater sensitivity in evaluating back pain, while the RDQ is most sensitive for patients with mild to moderate disability [19]. In terms of the NRS, the average difference between the second round of treatment and the initial round was −2.1, indicating a small difference. The decrease in the NRS indicates that the combined treatment of physical therapy and infiltration is beneficial for pain management. However, the small difference suggests that this scale lacks precision in describing low back pain, likely due to its nonspecific nature [18]. The literature supports the idea that both physical therapy and infiltration therapy are beneficial for managing low back pain. Additionally, combined therapy appears to be more effective, and ultrasound-guided infiltration allows for greater precision and better outcomes, as indicated by existing data [1]. Moderate-quality evidence suggests that home exercise programs [28], guided by physical therapists, can help lower the recurrence rate of acute back pain, extend the intervals between pain episodes, and reduce the reliance on healthcare services [29,30]. Intra- and extra-articular corticosteroid injections have been shown to provide pain relief for over three months in some individuals [31,32]. As this is a retrospective observational study, it is limited by the constraints of data availability and potential biases inherent in reviewing past records. Therefore, further studies are needed to explore the effects of combining physical therapy with an interventional approach on a larger population and with more specific protocols based on the degree of pain and functional limitation. Despite the need for additional research, the results we obtained indicate that combining physical therapy with an interventional approach is the best practice for managing low back pain.

## 5. Conclusions

This retrospective study suggests that combining ultrasound-guided infiltrations with early rehabilitation is effective in managing chronic nonspecific low back pain, and is more effective in reducing pain and disability in chronic low back pain compared to single interventions [33]. Further research is needed to develop targeted rehabilitation protocols and infiltrative methods for managing back pain. However, the combination of ultrasound-guided infiltration with early rehabilitation significantly reduces both pain and functional limitations in patients with low back pain.

## Figures and Tables

**Table 1:** Demographic characteristics (N = 40).

Age (average value)	41.5 years
Woman (*n*)	24
Men (*n*)	16
BMI (average value)	28.22
Manual worker (*n*)	22
Office worker (*n*)	18

**Table 2:** Oswestry Disability Index (ODI).

Variable	Comparison	Mean Difference	95% CI		*p*-Value
Time	Post1 vs. Pre	−18.8	−19.88	−17.72	<0.001
	Post2 vs. Pre	−30.0	−31.08	−28.92	<0.001
	Post2 vs. Post1	−11.2	−12.28	−10.12	<0.001
Sex	M vs. F	0.18	−0.89	1.24	0.758
BMI		0.02	−0.31	0.34	0.929
Age		−0.01	−0.09	0.07	0.832
Work	Office vs. Manual	−0.96	−2.00	0.09	0.094

Mean differences in ODI scores at different time points, showing a significant reduction in disability after each treatment cycle, with no significant differences related to sex, BMI, age, or work type.

**Table 3:** Quebec Back Pain Disability Scale (Qbpds).

Variable	Comparison	Mean Difference	95% CI		*p*-Value
Time	Post1 vs. Pre	−28.4	−36.35	−20.45	<0.001
	Post2 vs. Pre	−51.2	−59.15	−43.25	<0.001
	Post2 vs. Post1	−22.8	−30.75	−14.85	<0.001
Sex	M vs. F	3.81	−3.1	10.72	0.305
BMI		−0.21	−2.31	1.88	0.849
Age		0.22	−0.28	0.72	0.406
Work	Office vs. Manual	−2.41	−9.21	4.39	0.508

Significant improvements in QBPDS scores, particularly after the second cycle of treatment, with no significant differences based on sex, BMI, age, or work type.

**Table 4:** Roland Disability Questionnaire (RDQ).

Variable	Comparison	Mean Difference	95% CI		*p*-Value
Time	Post1 vs. Pre	−8.0	−8.74	−7.26	<0.001
	Post2 vs. Pre	−14.6	−15.34	−13.86	<0.001
	Post2 vs. Post1	−6.6	−7.26	−5.86	<0.001
Sex	M vs. F	0.19	−0.46	0.84	0.585
BMI		−0.11	−0.31	0.08	0.285
Age		0.03	−0.02	0.08	0.251
Work	Office vs. Manual	0.1	−0.54	0.74	0.767

Statistically significant reduction in RDQ scores across all time periods, with no differences linked to sex, BMI, age, or work type.

**Table 5:** Numeric Rating Scale (NRS).

Variable	Comparison	Mean Difference	95% CI		*p*-Value
Time	Post1 vs. Pre	−4.24	−4.66	−3.82	<0.001
	Post2 vs. Pre	−6.34	−6.76	−5.92	<0.001
	Post2 vs. Post1	−2.1	−2.52	−1.68	<0.001
Sex	M vs. F	0.09	−0.27	0.46	0.626
BMI		−0.03	−0.14	0.08	0.621
Age		0.01	−0.01	0.04	0.399
Work	Office vs. Manual	−0.02	−0.38	0.34	0.914

Significant reduction in pain levels as measured by the NRS, with the most notable improvement occurring after the second treatment cycle. No significant differences related to sex, BMI, age, or work type.

## Data Availability

The original contributions presented in the study are included in the article, further inquiries can be directed to the corresponding author.
